# Hemodynamic indicators of the formation of tandem intracranial aneurysm based on a vascular restoration algorithm

**DOI:** 10.3389/fneur.2022.1010777

**Published:** 2022-11-09

**Authors:** Yunchu Yao, Xin Tong, Yuqian Mei, Fan Yu, Yi Shan, Aihua Liu, Duanduan Chen

**Affiliations:** ^1^School of Life Science, Beijing Institute of Technology, Beijing, China; ^2^Department of Interventional Neuroradiology, Beijing Neurosurgical Institute and Beijing Tiantan Hospital, Capital Medical University, Beijing, China; ^3^School of Medical Imaging, North Sichuan Medical College, Nanchong, China; ^4^Department of Radiology and Nuclear Medicine, Xuanwu Hospital, Capital Medical University, Beijing, China; ^5^Beijing Key Laboratory of Magnetic Resonance Imaging and Brain Informatics, Beijing, China; ^6^School of Medical Technology, Beijing Institute of Technology, Beijing, China

**Keywords:** tandem intracranial aneurysms, hemodynamics, vortex core, growth sequence, vascular restoration algorithm

## Abstract

**Background:**

Hemodynamic factors are believed to be closely related to IA growth. However, the underlying pathophysiological mechanism that induces the growth sequence in tandem intracranial aneurysms (IAs) remains unclear.

**Methods and results:**

This study involved five patients with tandem IAs. Aneurysm models were reconstructed based on image datasets. A novel vascular restoration algorithm was proposed to generate the hypothetical geometry of the healthy parent vessel before each IA formation in the concatenated structure. Detailed hemodynamic patterns and morphological features were revealed under various growth sequences of tandem IAs to investigate the flow-driven mechanism of IA growth. Potential hemodynamic indicators of IA formation were proposed.

**Results:**

The patient cases were divided into two groups based on the size difference of tandem IAs. In the group with a similar size of tandem IAs, the position of the vortex core was associated with the site of the secondary aneurysm, while in the group with a significant size difference of the IAs, the position with the maximum curvature of the parent vessel plays a significant role in aneurysm formation.

**Conclusions:**

This study preliminarily revealed key hemodynamic and morphological indicators that determine the formation of tandem IAs. The proposed vascular restoration algorithm that provided the pre-aneurysm vasculature might be useful in investigating the flow-driven mechanism of IA growth, thus contributing to the risk evaluation of secondary aneurysm formation.

## Introduction

Intracranial aneurysms (IAs) are injuries or diseases of the intracranial arterial wall, resulting in localized or diffuse expansion or bulging of the wall. IAs occur in around 2–3% (1) of the general population, and the number keeps increasing due to the wide application of neuroradiological techniques (2, 3). Multiple or tandem IAs account for 15–35% of patients with unruptured IAs (4, 5). However, pathological factors leading to initiation, growth, and rupture of tandem IAs remain unclear (6–8).

To our knowledge, because of the low rupture rate (9) and the high risk of surgical complications, surgeons are in a dilemma over whether to treat the unruptured intracranial aneurysm. Clinical decisions must be considered to determine an aneurysm at a higher rupture risk. According to Jain's analysis of 18 cases with multiple ipsilateral aneurysms, the proximal aneurysm was more likely to rupture in 12 of the cases (10). Philipp Berg discovered that low WSS and complex flow structures occurred more frequently in ruptured aneurysms (11). Identifying the characteristics that trigger the start of the secondary aneurysm would contribute more accurately to determining the rupture risk of an aneurysm in the clinic. However, guidelines to determine the growth sequence of tandem aneurysms are warranted.

Computational fluid dynamics (CFD) can provide detailed hemodynamic information for the assessment of IAs and has become increasingly popular in IA research (12, 13). Among hemodynamic indicators, the standard time average characterization of wall shear stress (WSS) is defined as the time-averaged WSS (TAWSS) of the wall that correlates highly with IA inception (14). TAWSS has been proven to affect the biological process of the vascular wall, resulting in vascular remodeling. Mantha et al. proposed an aneurysm formation index (AFI) to describe the WSS vector direction changes over the cycle (15). Shimogonya et al. developed gradient oscillatory number (GON), which can quantify the degree of oscillating tension/compression forces in vascular wall (16). GON and AFI were detected to have a significant correlation with aneurysm formation (17).

Vortices were reported to alternate intravascular flow directionality on a broad scale and may lead to a pathological vasculature change, which is correlated with endothelial cell alignment (18, 19) and phenotypic expression (20, 21). J Xiang et al. analyzed 119 IAs by hemodynamic simulation and reported that complex flow patterns with multiple vortices were more frequently observed in ruptured aneurysms (61%). The hemodynamic indicators, such as TAWSS AFI, provided hemodynamic alterations at the vessel wall. Vortices were used to evaluate the gross flow complexity inside the vessel, but it might provide a new perspective, that is, surface hemodynamic parameters cannot be quantified (12).

This study included five patients with tandem IA, with separated IAs and the parent vessel's skeleton. We thoroughly investigated the possible preferential initiation of an aneurysm by using important hemodynamic parameters. Ultimately, the characteristics that might contribute to subsequent IA formation were summarized. To the best of our knowledge, this is the first study to determine the potential hemodynamic indicators that can indicate how a single aneurysm triggers the growth of the secondary one, which can enrich the results of previous studies and help surgeons identify risk factors that may cause tandem aneurysms.

## Materials and methods

### Creation of pre-aneurysm vasculatures

**Figure 1:**
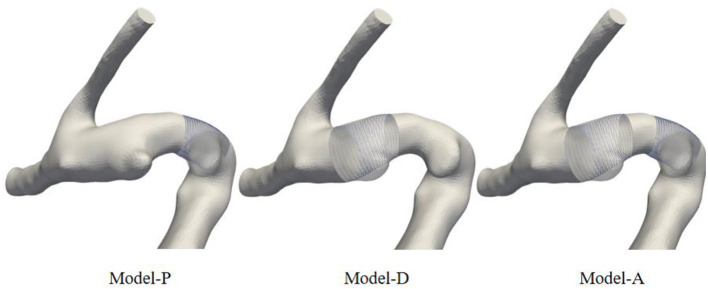
Schematic diagram of model comparison between pre-aneurysm vasculatures and the original model. The restored vessels were replaced by cylindrical tubes.

This study was approved by the Ethics Committee at Beijing Tiantan Hospital. This study involved five patients with tandem IAs. For each patient, CTA sequences were obtained from the clinic and imported into the Mimics Innovation suite for 3D segmentation. Mimics reconstructed vessel geometry using the image segmentation method based on signal intensity. The vascular restoration algorithm was used to create the pre-aneurysm vasculature. First, the original model, the skeleton, and the inflow and outflow cross-sections in the adjacent aneurysm area without diameter mutations were extracted. Next, the semi-automated algorithm was used to generate a cylindrical tube with a uniform diameter changing between the inflow and outflow sections. Three models were reconstructed: Model P, Model D, and Model A. One representative example of the original model and the results of 3 sets of pre-aneurysm vasculatures are shown in Figure 1. Model P represents the proximal aneurysm along with the parent vessel skeleton restored by a cylindrical tube, Model D represents the proximal aneurysm along with the parent vessel skeleton kept intact, and Model A represents all aneurysms removed.

### CFD simulation

The reconstructed aneurysm models were meshed in ICEM CFD and consisted of the tetrahedral grid in the core region and the prismatic grid near the vessel wall. Blood was simulated as an incompressible and Newtonian fluid with a dynamic viscosity of 0.0035 *Pa*·*s* and a mass density of 1,066 kg/m^3^. The vessel walls were assumed to be rigid walls with a non-slip boundary condition. The inlet and outlet boundary conditions were extracted from @neufuse software, where the database of human blood vessel boundary function information was contained. After simulating three cardiac cycles with 100 time steps each, 100 equally spaced time points were saved for data processing and analysis from the last cardiac cycle. The flow governing the Naviér-Strokes equation was solved by ANSYS CFX 19.2 (ANSYS Inc., Canonsburg, Pa) based on unstructured mesh.

### Extraction and analysis of significant parameters

TAWSS (14), AFI (15), GON (17), vortices (11), and the hemodynamic index for the incidence of IAs were calculated to quantitatively and qualitatively evaluate flow patterns in our research. Time-averaged WSS (TAWSS) is the standard time average of wall shear stress (magnitude) (22). AFI is defined as the cosine of the angle between the instantaneous WSS vector and the time-averaged WSS vector and can evaluate WSS vector direction changes over the cycle. GON is a non–dimensional parameter and quantifies the disturbance of the blood flow. GON depends on the degree of temporal fluctuations in the spatial WSSG vectors during a cardiac cycle. The Q-criterion was used to identify vortex regions where rotational flow dominated over straining flow. The vortex region was simply defined as *Q*>0, where positive Q indicates that the rotational flow occupies the main position in the flow domain (23, 24).

The above hemodynamic parameter expressions are shown in Table 1, where $\left|\overset{\bar{}}{\tau_{\omega }}\right|$ is the instantaneous WSS magnitude, T is the cardiac cycle period, τ_*i*_ is the WSS vector, τ_*av*_ is the time-averaged WSS *vector*, *G* = (∂*f*_*p*_/∂*p*, ∂*f*_*q*_/∂*q*) is the spatial wall shear stress gradient vector, S is the symmetric rate-of-strain tensor, and Ω is the antisymmetric rate-of-rotation tensor.

**Table 1:** The definition of hemodynamic parameters.

**Variable**	**Formula expression**	**Definition**
TAWSS	$TAWSS=\frac{1}{T}\overset{T}{\underset{0}{\int }}|\overset{\bar{}}{\tau_{\omega }}|\cdot dt$	Correlated with IA initiation
AFI	$AFI = cos\theta =\frac{\tau_{i}\times \tau_{av}}{\left|\tau_{i}|\times |\tau_{av}\right|}$	The risk of vortex intensity in stagnant areas due to the formation of aneurysms
GON	$GON=1-\frac{\left|\overset{T}{\underset{0}{\int }}Gdt\right|}{\overset{T}{\underset{0}{\int }}|G|dt}$	Fluctuations in the tension/ compression force acting on endothelial cells
vortices	$Q\equiv \frac{1}{2}\left(\Omega^{2}+S^{2}\right)$	A balance between shear strain rate and vorticity magnitude (25)

### Regions of interest

**Figure 2:**
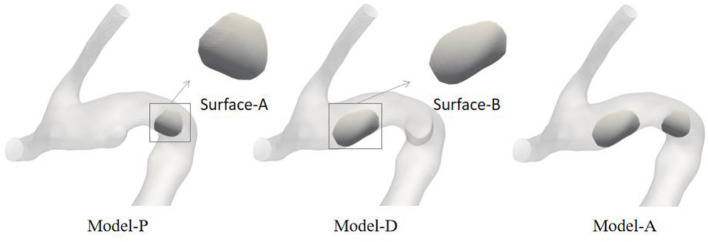
Schematic diagram of the ROI of three different series of pre-aneurysm vasculatures.

To quantitatively evaluate the changes in hemodynamic parameters between different areas containing IA inception, areas that contained aneurysm occurrence of each model were chosen based on the regional positioning algorithm. First, the artificially selected aneurysm neck plane was used as the positioning plane, and the pre-aneurysm vasculatures were projected onto the neck plane to determine the area of the aneurysm inception. Next, the IA inception region was localized in the partial area according to the principle that the angle between points in the aneurysm neck curve and each point in the IA inception region is equal to 360°. These points were imported into the Geomagic Studio 2012 (64-bit) software and converted into surfaces. One representative example of the ROI region in three sets of pre-aneurysm vasculature is shown in Figure 2. Surface-A represents the region of proximal aneurysm initiation, which is different from Surface-B, presenting the region of distal aneurysm initiation.

## Results

### Morphological parameters analysis

To further investigate the possible causes of IA initiation, the morphological parameters that could reflect the degree of curvature of the models, such as curvature and DM, were extracted and analyzed. Curvature is a parameter of the degree of the curve deviating from the straight line; DM is the ratio of the straight-line distance to the curve distance between the start point and the endpoint. The skeletons of pre-aneurysm vasculatures were extracted from all patients to extract morphological parameters. Additionally, the comparison of morphological parameters to different groups of the models can be seen in Table 2, which shows the lack of a strong correlative relation between IA inception and the morphological parameters of the pre-aneurysm vasculatures. The different-sized groups showed a higher DM value than the same-sized group, but it was insignificant.

**Table 2:** The summary of the comparison of morphological parameters of different pre-aneurysm vasculatures.

**Morphological parameters**					
		**mean_curvature**	**max_curvature**	**min_curvature**	**DM**
Same szie	Case 1	0.133	0.729	0.001	0.595
	Case 2	0.194	2.363	0.012	0.371
	Case 3	0.177	0.731	0.006	0.533
Size difference	Case 4	0.121	0.610	0.003	0.612
	Case 5	0.137	0.871	0.008	0.622

Color values indicate the location of the maximum or minimum parameter.

**Figure 3:**
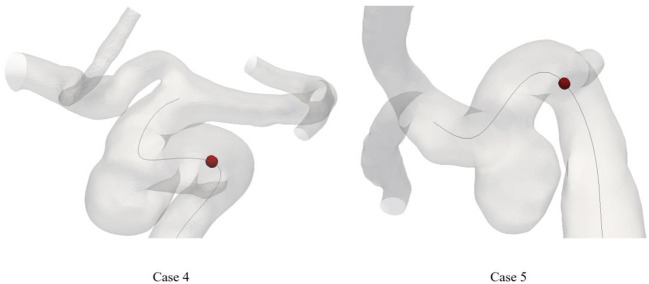
The location of maximum curvature point on skeleton of patient. The dark line means the skeleton. The maximum curvature point was plotted as a red sphere.

Considering the correlation between curvature and model specificity, we performed a secondary analysis of the maximum curvature position of all patients. Figure 3 shows the position of the maximum curvature point relative to the skeleton. The black line in Figure 3 indicates the model skeleton. Additionally, the red sphere in the black line represented the maximum curvature position of the patient. Coincidentally, the point of maximum curvature corresponded to the area of IA incidence in the second group. In contrast, the same pattern was not found in the first group.

### Quantitative and qualitative hemodynamic parameters analysis

**Figure 4:**
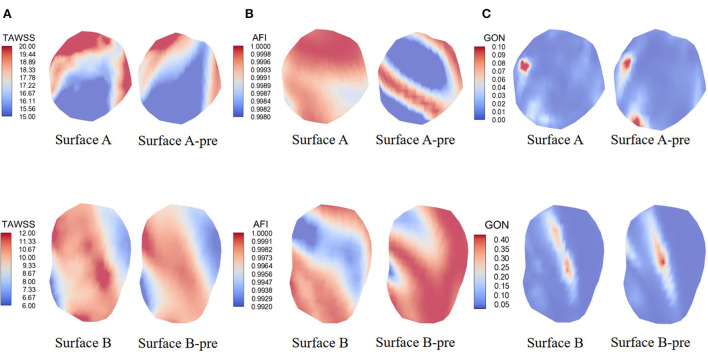
Hemodynamics map comparison of two ROI presenting significant characteristics. **(A–C)** showed the distribution of TAWSS, AFI, and GON. Surface A represents the hemodynamic changes in the proximal aneurysm growth area when the distal aneurysm keeps intact; Surface B represents the hemodynamic changes in the distal aneurysm growth area when the proximal aneurysm keeps intact; Surface A-pre represents the hemodynamic changes in proximal aneurysm growth area when the distal aneurysms were removed; Surface B-pre represents the hemodynamic changes in distal aneurysm growth area when the proximal aneurysms were removed.

Hemodynamic calculations were performed on all pre-aneurysm vasculatures, and hemodynamic parameters are drawn in Figure 4. TAWSS, AFI, and GON distributions are shown in Figures 4A–C. Surface A (B) represented the hemodynamic changes in the proximal (distal) aneurysm inception area, respectively, when the distal (proximal) aneurysm was kept intact. Surface A-pre(B-pre) represents the hemodynamic changes in the proximal (distal) aneurysm inception area when both aneurysms were removed. The differences in the changes between Surface A-pre and Surface A, Surface B-pre, and Surface B were compared, which were used to simulate the hemodynamic parameter variation of region of interest (ROI) affected by the growth of another aneurysm in different aneurysm growth sequences. Figure 4A displayed the TAWSS distribution, and the color bar was restricted in the range of 15–20 and 6–12 Pa to reflect the comparison between different growth sequences. Compared with Surface B, Surface A had a slight increase in the high TAWSS area.

AFI and GON are two WSS-derived parameters invented to explore the risk of aneurysm formation. Figure 4B shows the mid-diastolic AFI distribution. The color bars were restricted to 0.998 to 1 and 0.992 to 1 units to reflect AFI distribution under different growth states. It was explicitly shown that Surface A presented an increased risk of low AFI when another aneurysm grew. However, Surface B had the opposite change. Figure 4C shows the GON distribution of the indifferent IA initiation region. Opposite changes were also exhibited on both surfaces. Surface A displayed a decrease in the peak GON regions, but Surface B displayed an increase in the peak regions.

As shown in Figure 4, the resultant gradient variation was affected by the maximum and minimum values of the color bar, which might affect the subsequent analysis results. The inception region of the aneurysm is interpolated as a collection of tiny two-dimensional triangular fragments of equal length on the sides. The hemodynamics parameter values in the same IA initiation region were blended, and the maximum and minimum values were extracted. Variable ranges were divided into four grades by the peaks and valleys. The area of the highest grade parameters was calculated according to the triangle area fragments. The same method was applied to calculate the variable of the area-changing ratio of hemodynamic parameters. The calculated parameters are displayed in Table 3.

**Table 3:** The comparison of the area-changing ratio of hemodynamic parameters of different pre-aneurysm vasculatures.

**Patient case**				
Same size			Model-P	Model-D(remote)
	Case 1	TAWSS	1.000	0.086
		AFI	0.785	−0.222
		GON	0.604	−0.583
			Model-P	Model-D(remote)
	Case 2	TAWSS	0.902	1.551
		AFI	−0.007	0.004
		GON	1.727	−0.516
			Model-P	Model-D(remote)
	Case 3	TAWSS	0.112	−0.433
		AFI	−0.076	−0.200
		GON	0.490	−0.423
Size difference			Model-P	Model-D(remote)
	Case 4	TAWSS	−0.006	2.943
		AFI	−0.026	−0.436
		GON	0.537	1.000
			Model-P	Model-D(remote)
	Case 5	TAWSS	0.144	0.233
		AFI	0.029	−0.017
		GON	5.745	−0.873

The colored cells indicate the priority of aneurysm initiation.

Combined with previous studies, we considered negative AFI with a higher absolute value, positive GON, and TAWSS with a higher value to be risk factors inducing initiation of secondary IA (15, 17, 26). When the hemodynamic parameter change in Surface A of Model-P displayed a higher area-changing ratio of TAWSS compared with Surface B of Model-D, this study assumed that Model-P could be concluded as the possible preferential initiation model under the current hemodynamic parameters. The analysis method was similar for other hemodynamic parameters, and the above analysis procedure was performed on pre-aneurysm vasculature. There was a low probability that three hemodynamic parameters pointed to the same possible preferential initiation models. When two or more hemodynamic parameters were pointed to the same model, the model was considered as the possible preferential initiation model. The possible preferential initiation models are highlighted in Table 3. Aneurysms in the five cases were divided into two groups according to the morphology size of both IAs. In most models, the area-changing ratio on the ROI had a significant variation when one IA grew, which proved that the incidence of one IA might contribute to the growth of another aneurysm.

### Vortex core analysis

**Figure 5:**
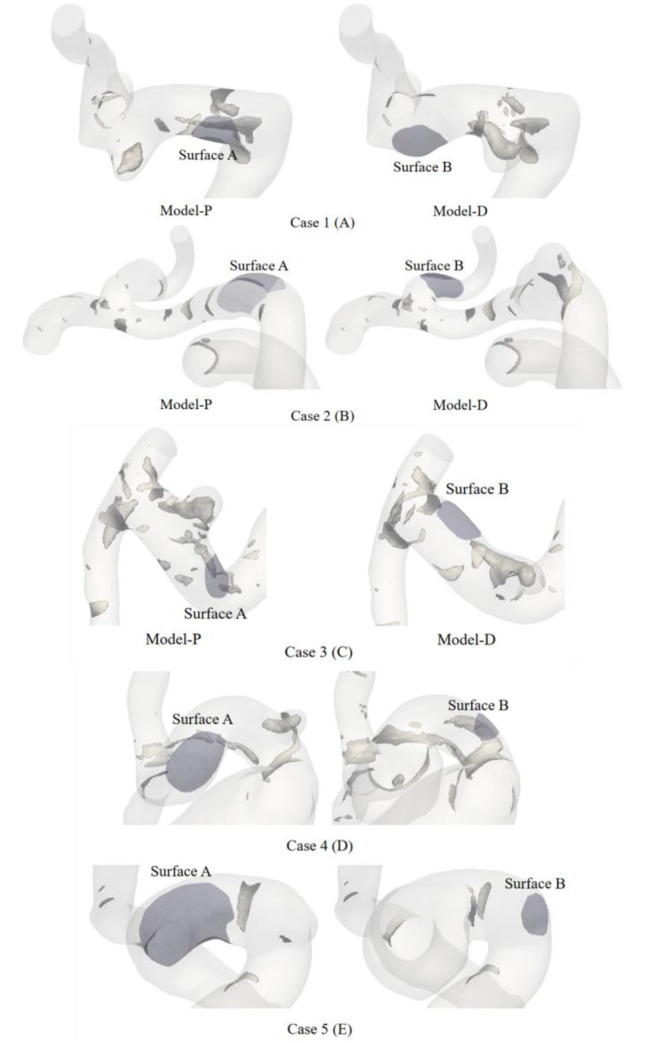
The hyaline body is the vessel, the tubular gray bodies clustered on the surface of the vessels are the vortex core. Vortex core in the parental intravascular region was recognized by positive Q-criterion. The violet area in each case presents the aneurysm ROI area.

The vortex core is an important manifestation of the vortex flow, which could offer a key vehicle for describing an aneurysmal flow field in the respects such as location. The vortex core was extracted by Q-criterion with a given threshold value of Q, which could provide a direct description of the vortex. This study set the same threshold to extract the vortex core for each case. The visualization results of the vortex core of a typical case are displayed in Figure 5A. Regarding view consistency, the possible preferential initiation models were placed in the left graph. The right-hand side of the graph was filled with models contrary to our judgment. In addition, the dark area in each case represents the aneurysm ROI area.

The possible preferential initiation model in the left graph appeared to have a limited number of cores. The structures of the intra-vascular vortex core were shaped like a small tube, gathering near Surface A in Figure 5A. Meanwhile, no vortex core could be extracted around Surface B. The vortex cores were extracted from the other four patients and are presented in Figures 5B–E. Figures 5B,C showed the same phenomenon as Figure 5A. However, only a small and flaky vortex core region was found in Model-P in Figure 5B. Figure 5D presented obvious vortex cores in the IA initiation region of both growth sequence models. Relatively, Model-P showed a more distinct vortex core in Figure 5D. Inversely, Figure 5E showed no obvious vortex cores in the IA initiation region of different growth sequence models.

## Discussion

In this study, the variation of hemodynamic indices in the IA initiation regions was inconsistent with different patient models. Preliminary, limited hemodynamic parameters were included in our study to ensure the accuracy of estimating a possible IA growth priority model. In this study, we attempted to evaluate the flow complexity inside the vessel by adding an additional hemodynamic indicator vorticity core to provide a unique perspective for the study of aneurysm initiation. Collectively, this study provided ideas for evaluating tandem aneurysms' inception and treatment by analyzing hemodynamic factors.

The TAWSS, GON, and AFI were gathered as a parameter group and quantified the changing percent in the IA inception region under two different growth sequences. The adoption regulation of these parameters limited the indices into parameter groups because they have been extensively researched and proved to be correlated with the IA inception region and could impact IA initiation (11, 14, 15, 17). The previous study pointed out that OSI and AFI had a high correlation (11). The OSI was removed from the study. Table 2 shows that the area-changing ratio contributing to the aneurysm initiation significantly changed which aneurysm growth occurred first, which indicates an obvious impact. The growth of one aneurysm triggered the growth of a secondary aneurysm was the acceptable hypothesis in this study. The possible IA growth priority model was explored based on preliminary results. There were proximal aneurysms and distal aneurysms among the pre-growth aneurysms. The hypothesis that the location of aneurysms in the same vessel could be used as a determinant for a single aneurysm triggering the subsequent aneurysm growth is untenable. If the order of temporal occurrence for IAs was known, further discussion could be carried out.

To ascertain the relevance of vorticity to aneurysm inception, we extracted a three-dimensional vortex core based on the Q-criterion in all the pre-aneurysm vasculatures. In previous studies, vortex cores have been widely used to explore the relationship between IA rupture and hemodynamic characteristics. To compare vortex structures across a large number of patient cases, Nicole Varble used vVF and sVF to measure the prevalence of vortical flow in each aneurysm (24). Kevin Sunderland proved the degree of vortex overlap (DVO) to measure the temporal stability and the [normalized] $\overset{}{Q}$ and $\overset{}{\lambda_{2}}$ values replacing the original *Q* and λ_2_ values to condense the distributions vortex cores region (27). In our research, 8% was used as a given threshold of positive peak value Q to reduce the sizes of the identified vortex core regions at the peak systolic period. The low value significantly reduced the sizes of identified vortex core regions and contributed to determining the location of the vortex core.

In the current study, we found that the ROI of IA initiation was associated with a positive Q vortex core near the wall (Figure 4) of the first group of patients. Previous studies reported the relation between vortical flow and IA rupture (12, 28–31). The presence of the vortex core indicates alternate directions of intravascular flow, changing phenotypic expression and endothelial cell alignment, which might lead to vasculature pathological changes (18–20, 32). Sunderland proved that combining the mean number of vortices (MV) for predicting IA inception was the strongest prediction model (11). Our conclusions were consistent with the conclusions by Sunderland that the ROI region corresponded to the vortex core in the possible IA growth priority model we estimated with AFI. The vortex core could activate mural-cell-mediated destructive remodeling mechanisms and could act as a possible judgment factor for the incidence of aneurysms (24). However, standardized methods for determining the vortex core region among a large number of patients need to be addressed in future research, which might influence the accuracy of the result.

From the second set of patients, we were able to isolate the disordered vortex core or the absence of a vortex core. This is to be expected after the maximum curvature position in relation to the skeleton was identified. The pre-growth aneurysm incidence region corresponded to the position of the maximum curvature point. Lauric noted that a larger curvature indicator may result in a dramatic elevation of WSS and WSS gradient magnitude (33). This result explains our previous hypothesis for the possible IA growth priority model of the tandem aneurysm.

The morphological factors of ruptured aneurysms have always been a reference factor for clinical interventional treatment. However, the effect of aneurysm size on aneurysm rupture has been controversial. Previous studies showed that larger aneurysms have a higher risk of rupture (34). A growing number of studies proved the opposite conclusion. Daan Backes showed that, in one-third of the patients with aneurysmal SAH and multiple intracranial aneurysms, the ruptured aneurysm was not the largest aneurysm (35).

Meanwhile, Hai-Tao Lu found that the majority of the ruptured aneurysms were small (68%) (36). In this study, the order of temporal occurrence for multiple IAs may provide a new way to study aneurysm rupture. The smaller aneurysm near the high curvature position grew first, and the larger aneurysm was initiated based on abnormal hemodynamics caused by the small aneurysm. Smaller aneurysms have a higher risk of rupture and may be related to pathological conditions triggering degenerative remodeling for a long time. This might lead to the smaller aneurysms having an unstable artery wall and being more likely to rupture.

Due to the scarcity of the parental vascular model before aneurysm growth, the vascular restoration method occupied our research's main position. Sunderland et al. proposed a method for restoring the vessels' lumen by VMTK in 2019 (11). Although this approach could be well applied at the bifurcation, the inevitable error would lead to unintended alterations to parent vessel curvature, which is crucial in the error of hemodynamic calculation on aneurysm reduction. Sunderland et al. resolved this error by manual modification. To solve this problem, a practical algorithm has been proposed for digitally removing an aneurysm from the parent artery in this study (11). The vascular restoration algorithm only changed the aneurysm initiation region and retained the parental vessel characteristics. It used 3D tubes with uniformly varying diameters instead of aneurysms, which were also applied at the bifurcation. However, as in the method described in the study by Sunderland et al., only two major vessels were preserved. Ultimately, the local area of the aneurysm growth was extracted. Chen et al. (37) compared hemodynamic results in the flake region, which are clear and could be updated. The ROI in the region of IA initiation was located to improve the reliability of the dataset in our research, avoiding the influence of the hemodynamic parameters of the invalid area.

There were several limitations to this study. First, the patient types are similar, so a selection bias may exist. Second, the patient-specific velocity waveforms could not be obtained from the hospital. To simulate the pulsating CFD, we used the database of human blood vessel boundary function information instead. Although the vortex core was proved to be the judgment factor for second aneurysm growth in our research, future research needs to expand the sample size and enrich the sample. Finally, although the simple Q-criterion was adopted in our research, there are many other definitions of the vortex, including the λ criterion and so on (23). In future studies, different methods of vortex core definition can be used to provide additional insights into the behavior of vortex cores in IAs.

## Conclusion

In our research, the possible preferential initiation models were estimated by changing hemodynamic parameters. The ROI of IA initiation was located to improve the accuracy of the dataset. The growth of one single aneurysm significantly affects the hemodynamics of another aneurysm, suggesting a possible IA growth priority model for tandem aneurysms. Ultimately, obvious vortex cores were extracted in the IA incidence region in the same size group. Although similar vortex core information was not found in the different size groups, the point of maximum curvature was found to correspond with the area of the IA incidence. The vortex core information might guide tandem aneurysms to induce the growth of the subsequent aneurysm. The order of temporal occurrence for multiple IAs and vortex cores may provide a new perspective to studying aneurysm rupture. The assumption that smaller aneurysms have a higher risk of rupture and an unstable artery wall was proposed because of the pathological conditions triggering degenerative remodeling for a long time.

## Data availability statement

The original contributions presented in the study are included in the article/Supplementary material, further inquiries can be directed to the corresponding authors.

## Ethics statement

The studies involving human participants were reviewed and approved by Ethics Committee at Beijing Tiantan Hospital. The patients/participants provided their written informed consent to participate in this study. Written informed consent was obtained from the individual(s) for the publication of any potentially identifiable images or data included in this article.

## Author contributions

DC and AL designed the research studies. YY, YM, FY, and YS collected the patient data and analyzed the data. YY and XT wrote the manuscript. All authors contributed to the article and approved the submitted version.

## Funding

This work was supported by Beijing Natural Science Foundation (Z190014 and L192010).

## Conflict of interest

The authors declare that the research was conducted in the absence of any commercial or financial relationships that could be construed as a potential conflict of interest.

## Publisher's note

All claims expressed in this article are solely those of the authors and do not necessarily represent those of their affiliated organizations, or those of the publisher, the editors and the reviewers. Any product that may be evaluated in this article, or claim that may be made by its manufacturer, is not guaranteed or endorsed by the publisher.
