# Supplementary material for: Hemodynamic indicators of the formation of tandem intracranial aneurysm based on a vascular restoration algorithm
Source: Front Neurol. 2022 Nov 9;13:1010777. doi: 10.3389/fneur.2022.1010777 (PMC9683096; doi:10.3389/fneur.2022.1010777)
Supplement: Supplementary file 1 [file Image_1.pdf]

Supplemental Information

Supplementary figure showed the hemodynamic images of all the remaining patient. The color bars of parameters were restricted to different ranges to reflect the distribution of parameters in different growth states. As for AFI, surface A presented the increase of low AFI when another aneurysm grew in case 4 while the opposite change could be found in case 5. The changes in surface B are not obvious in case 2, 3 and 5. In case 3, surface B presented the increase of low AFI when another aneurysm grew. As for GON, opposite changes were also exhibited on both surfaces. In case 5, Surface A displayed the decrease of the peak GON regions but Surface B displayed the increase of the peak regions. In other cases only partial surface changes could be observed. As for the TAWSS distribution in case2, Surface B and surface A had a slight increase in the high TAWSS area. The surface B in case 3 had an obvious trend of decreasing area of high value area. Surface B presented the increase of low TAWSS when another aneurysm grew in case 3 while the opposite change could be found in case 2. In most cases, it is difficult to assess the exact value of change.

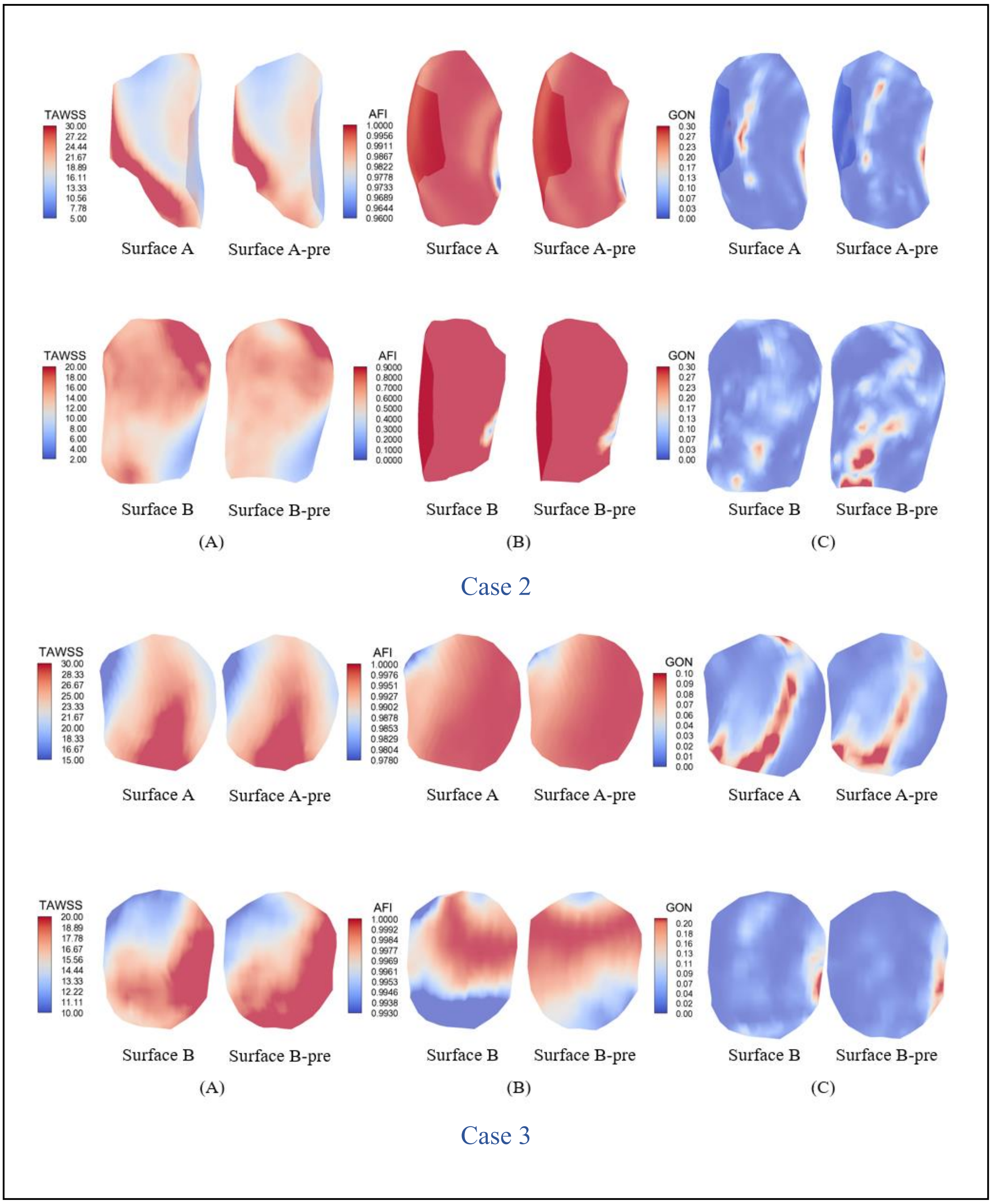

TAWSS
30.00
27.22
24.44
21.67
18.89
16.11
13.33
10.56
7.78
5.00
AFI
1.0000
0.9956
0.9911
0.9867
0.9822
0.9778
0.9733
0.9689
0.9644
0.9600
GON
0.30
0.27
0.23
0.20
0.17
0.13
0.10
0.07
0.03
0.00
Surface A
Surface A-pre
Surface A
Surface A-pre
Surface A
Surface A-pre
TAWSS
20.00
18.00
16.00
14.00
12.00
10.00
8.00
6.00
4.00
2.00
AFI
0.9000
0.8000
0.7000
0.6000
0.5000
0.4000
0.3000
0.2000
0.1000
0.0000
GON
0.30
0.27
0.23
0.20
0.17
0.13
0.10
0.07
0.03
0.00
Surface B
Surface B-pre
Surface B
Surface B-pre
Surface B
Surface B-pre
(A)
(B)
(C)
Case 2
TAWSS
30.00
28.33
26.67
25.00
23.33
21.67
20.00
18.33
16.67
15.00
AFI
1.0000
0.9976
0.9951
0.9927
0.9902
0.9878
0.9853
0.9829
0.9804
0.9780
GON
0.10
0.09
0.08
0.07
0.06
0.04
0.03
0.02
0.01
0.00
Surface A
Surface A-pre
Surface A
Surface A-pre
Surface A
Surface A-pre
TAWSS
20.00
18.89
17.78
16.67
15.56
14.44
13.33
12.22
11.11
10.00
AFI
1.0000
0.9992
0.9984
0.9977
0.9969
0.9961
0.9953
0.9946
0.9938
0.9930
GON
0.20
0.18
0.16
0.13
0.11
0.09
0.07
0.04
0.02
0.00
Surface B
Surface B-pre
Surface B
Surface B-pre
Surface B
Surface B-pre
(A)
(B)
(C)
Case 3

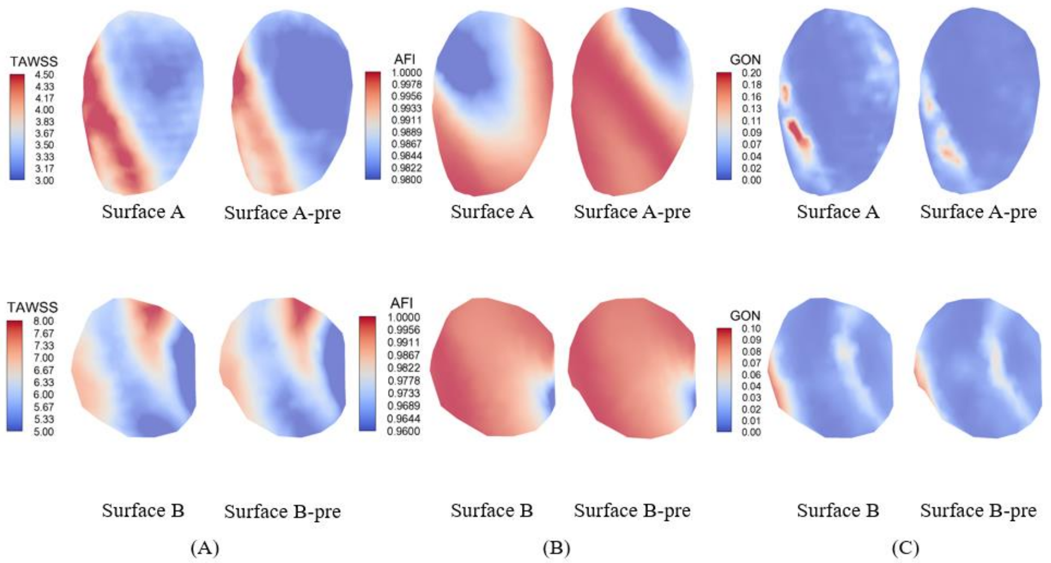

Case 4

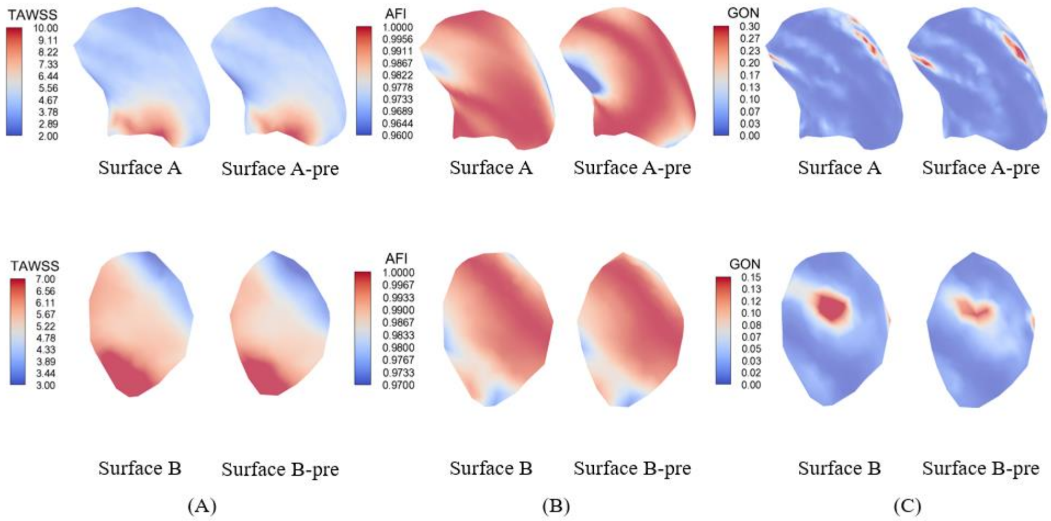

Case 5

Fig. Hemodynamics map comparison of two ROI presenting significant characteristics. (A-C) showed the distribution of TAWSS AFI and GON.

*Surface A represents the hemodynamic changes in the proximal aneurysm growth area when the distal aneurysm keeps intact; Surface B represents the hemodynamic changes in the distal aneurysm growth area when the proximal aneurysm keeps intact; Surface A-pre represents the hemodynamic changes in proximal aneurysm growth area when the distal aneurysms were removed; Surface B-pre represents the hemodynamic changes in distal aneurysm growth area when the proximal aneurysms were removed.
